# Cardiac Gated Computed Tomography Used to Confirm Iatrogenic Aortic Valve Leaflet Perforation after Mitral Valve Replacement

**DOI:** 10.1155/2013/528439

**Published:** 2013-03-04

**Authors:** Luke Oakley, Kathleen Love, Alfredo Ramirez, Gilbert Boswell, Keshav Nayak

**Affiliations:** ^1^Department of Internal Medicine, Naval Medical Center San Diego, Suite 301, 34800 Bob Wilson Drive, San Diego, CA 92101, USA; ^2^Department of Cardiology, Naval Medical Center San Diego, San Diego, CA 92101, USA; ^3^Department of Cardiothoracic Surgery, Naval Medical Center San Diego, San Diego, CA 92101, USA; ^4^Department of Radiology, Naval Medical Center San Diego, San Diego, CA 92101, USA

## Abstract

Aortic insufficiency from iatrogenic valve perforation from nonaortic valve operations is rarely reported despite the prevalence of these procedures. Rapid diagnosis of these defects is essential to prevent deterioration of cardiac function. In this paper, we describe a young man who reported to our institution after two open cardiac surgeries with new aortic regurgitation found to be due to an iatrogenic perforation of his noncoronary aortic valve cusp. This defect was not appreciated by previous intraoperative transesophageal echocardiography and was inadequately visualized on follow-up transthoracic and transesophageal echocardiograms. In contrast, cardiac gated computed tomography clearly visualized the defect and its surrounding structures. This case highlights the utility of cardiac gated computed tomography for cases of suspected valvular perforation when echocardiography is not readily available or inadequate imaging is obtained.

## 1. Introduction

 Aortic insufficiency resulting from iatrogenic aortic valve perforation during nonaortic valve cardiac operations is a rarely reported complication despite the prevalence of these procedures. Rapid diagnosis and treatment of these defects are essential in preventing deterioration of cardiac function.

This case report describes a young man with a history of mechanical mitral valve placement in 2004 and reoperation at an outside facility in 2009 secondary to prosthetic valve endocarditis who presented to our institution two months after the second procedure with progressive dyspnea and decompensated heart failure. He was found to have new severe aortic regurgitation from an iatrogenic perforation of his noncoronary aortic valve cusp.

Intraoperative transesophageal echocardiography (TEE) is established as an effective modality to detect these injuries during valvular replacement surgeries but in this case failed to detect a defect. Making the diagnosis involved use of transthoracic echocardiography (TTE), repeat TEE, and cardiac gated computed tomography (CT) to fully characterize the defect. This case highlights the diagnostic utility of cardiac CT in cases of new valvular insufficiency when iatrogenic injury is suspected.

## 2. Case Presentation

In September 2004, a 24-year-old male presented with progressive dyspnea on exertion, paroxysmal atrial fibrillation, malaise, and peripheral eosinophilia. Initial evaluation showed a left atrial mass and a separate mass adherent to the mitral valve for which the patient underwent surgical resection and mechanical mitral valve replacement. The diagnosis of noninfectious hypereosinophilic granulomatous disease was made by histopathologic evaluation. The patient underwent treatment with imatinib mesylate and a 5-month steroid course, which eventually led to complete resolution of eosinophilia, residual masses, and symptoms.

In December of 2009, the patient presented to an outside facility with fevers, night sweats, and weight loss and was diagnosed with subacute mechanical valve endocarditis from *Streptococcus* viridians. TTE noted multiple large vegetations at the four and five o'clock positions of the mechanical valve. The patient underwent a second mitral valve replacement using a 29 mm St. Jude mechanical valve, with a cross-clamp time of 85 minutes and total bypass time of 132 minutes. Intra-operative TEE was performed without mention of aortic valve injury or regurgitation. Pathology review of the prosthetic valve vegetations showed focal necrosis with fibrinous exudates without prominent eosinophilia. A two-day postoperative TTE report noted new mild to moderate aortic insufficiency. His postoperative course was significant for atrial flutter and mild congestive heart failure symptoms. He was discharged on appropriate heart failure therapy and neurohormonal blockade.

**Figure 1:**
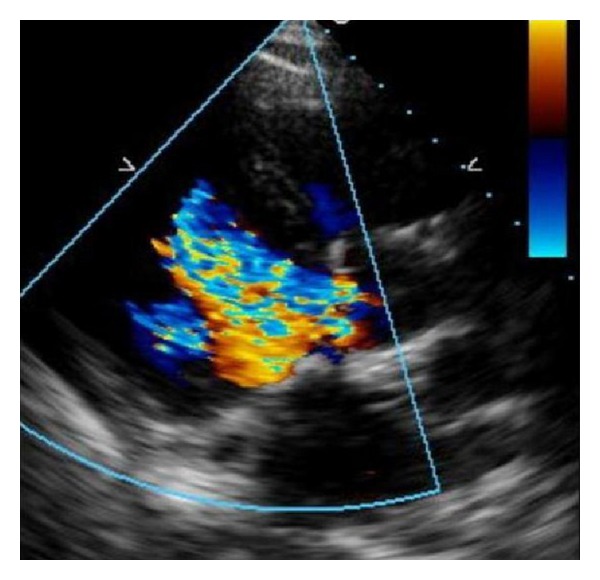
Transthoracic echocardiogram. Parasternal long-axis view reveals aortic regurgitation with both central and peripheral jets suggestive of valve perforation.

The patient's condition continued to deteriorate, and he presented to our institution 2 months after the second operation with NYHA Class III decompensated heart failure. Repeat echocardiography revealed an overall preserved ejection fraction of 45%–55%; however, a new finding of severe aortic regurgitation was noted (Figure 1).

**Figure 2:**
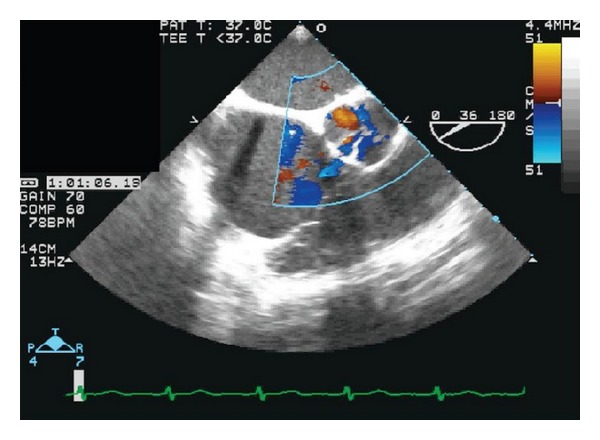
Transesophageal echocardiogram. Short-axis view shows more clearly the perforation and regurgitant jets. See Supplementary CINE file in Supplementary Material available online at http://dx.doi.org/10.1155/2013/528439.

TEE also demonstrated a large regurgitant jet originating from the non-coronary and left coronary cusps concerning for valve perforation (Figure 2).

**Figure 3:**
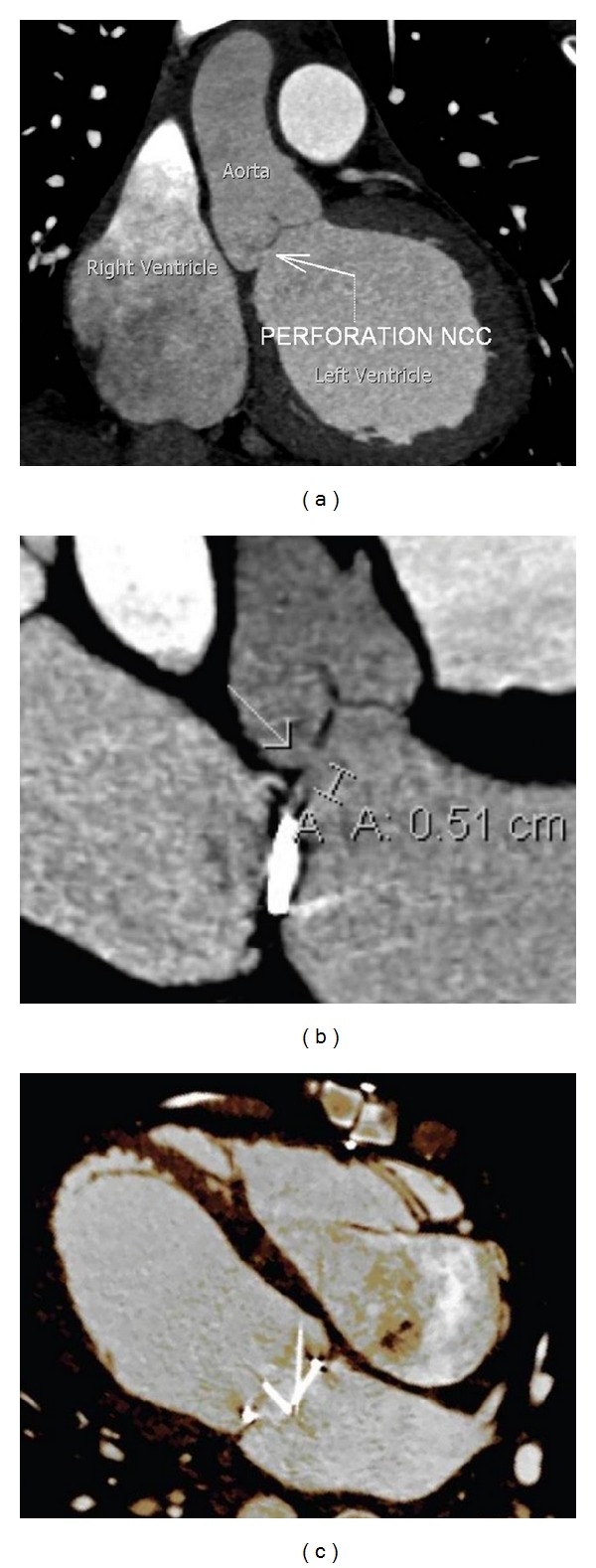
Cardiac gated computed tomography. (a) Large and (b) magnified oblique reformatted images of the left ventricular outflow tract and aortic valve demonstrate a clearly visible 5 mm defect in the non-coronary cusp of the aortic valve. (c) Four-chamber orientation shows the asymmetric movement of the bileaflet mechanical mitral valve due to the regurgitant jet originating from the aortic valve defect. Supplementary CINE file online shows 3D rendering of paradoxical mitral valve motion (see supplementary CINE file).

For better visualization, a cardiac gated CT scan was done, which confirmed a 4-5 mm perforation of the aortic valve non-coronary cusp (Figure 3).

Given extensive scarring from his recent surgical procedures, the patient underwent a trial of optimal medical management for the ensuing 4 months. Despite these efforts, he did not recover and was referred for surgical correction requiring a third sternotomy. Operative evaluation revealed a misplaced prolene suture used in closure of the transseptal surgical approach which perforated the non-coronary cusp. The defect was repaired with a pledgeted subannular stitch to reapproximate the leaflet. Intra-operative TEE demonstrated aortic and tricuspid valve competence, without stenosis or insufficiency. Postoperatively the patient made an excellent recovery.

## 3. Discussion

Aortic regurgitation resulting from leaflet perforation is typically the result of infectious endocarditis rather than iatrogenic injury [1]. However, due to its central location, injury to the aortic valve can occur during many cardiac procedures. Such injury has been most commonly reported as a result of accidental suture placement or laceration by a needle with sutures placed near the aortic annulus but has also occurred during guide-wire passage and from tension on the leaflet intraoperatively [2, 3]. Though iatrogenic injury to the aortic valve is rare, it is most commonly associated with procedures involving the mitral valve given its anatomic proximity to the non-coronary and left coronary cusps. As the aortic valve is typically not accessible for direct visualization during a left atrial approach, it can be subject to unvisualized errors [2].

Intra-operative TEE is now the standard imaging modality during valvular operations to confirm valvular integrity. Despite its use in this case, iatrogenic injury was not appreciated resulting in severe and debilitating consequences for the patient. Postoperatively, the diagnosis of valvular perforation has been made by visual inspection during follow-up surgery, TEE, and three-dimensional TEE [2, 4–8]. Typical echocardiographic findings include echo dropout in the body of the cusp, and an eccentric regurgitant jet. Often the short-axis view can confirm that the regurgitant flow originates from a perforation in the body of the cusp, rather than from a central or commissural location [2]. In their review of 3 D TEE on this subject, Thompson et al. suggest that this newer modality may provide added benefit when compared to traditional 2 D echocardiography, although no perforations in their study were iatrogenic in etiology [8].

Accurate diagnostic information is crucial in planning the method of repair and minimizing time spent on cardiopulmonary bypass. Repair of these lesions can be done by release of the offending stitch, which may compromise the prior intervention, repairing the valve, or valve replacement. Rother et al. described a case of suture-induced aortic valve perforation found intraoperatively which was not amenable to repair even after the offending suture was cut, thus forcing replacement of the aortic valve [9]. In the case described herein, the valve was amenable to repair months after the original operation. In less-severe cases, surgery may not be required with continued close monitoring and medical management [7].

To our knowledge, this is the first case to utilize cardiac gated CT to confirm the diagnosis of iatrogenic valve perforation and aid in surgical planning. This modality, relatively new compared with traditional echocardiography, is increasingly being used to characterize valvular pathology when inadequate images are obtained with other noninvasive methods and now has updated appropriateness guidelines favoring its use for evaluation of both native and prosthetic valve pathology [10]. In our case, TEE suggested that the regurgitant jet originated from within the cusp, but cardiac CT was better able to identify the size and position of the defect in three dimensions.

## 4. Conclusions

Aortic insufficiency resulting from iatrogenic aortic valve perforation may be an underreported complication, which can occur with both open and catheter-based procedures. This case illustrates an unusual presentation of heart failure in post-operative patients and highlights some of the challenges encountered when making the diagnosis. Cardiac gated CT is a feasible and appropriate alternative for diagnostic evaluation if TEE is not readily available or inadequate imaging is obtained.

## Supplementary Material

Transesophageal echocardiogram. Short-axis view demonstrates a large aortic regurgitant jet, as suggested by the orange doppler signal over the non-coronary cusp during diastole (Supplementary Material 1). Cardiac gated computed tomography with a reformated four-chamber view shows how the oblique regurgitant jet (not visualized) from the aortic valve leads to asymmetric motion of the bileaflet mechanical mitral valve (Supplementary Material 2).

## Abbreviations

TEE: Trans-esophageal echocardiography
TTE: Transthoracic echocardiography
CT: Computed tomography.
